# Associations between biopsychosocial factors and chronic upper limb pain among slaughterhouse workers: cross sectional study

**DOI:** 10.1186/s12891-016-0953-7

**Published:** 2016-02-27

**Authors:** Emil Sundstrup, Markus D. Jakobsen, Mikkel Brandt, Kenneth Jay, Per Aagaard, Lars L. Andersen

**Affiliations:** National Research Centre for the Working Environment, Lersø Parkalle 105, Copenhagen, Denmark; Institute for Sports Science and Clinical Biomechanics, SDU Muscle Research Cluster (SMRC), University of Southern Denmark, Odense, Denmark; Physical Activity and Human Performance Group, Center for Sensory-Motor Interaction, Department of Health Science and Technology, Aalborg University, Aalborg, Denmark; Institute for Sport and Clinical Biomechanics, Department of physical activity and health, University of Southern Denmark, Odense, Denmark

**Keywords:** Rapid force capacity, RFD, PPT, Shoulder pain, Arm pain, Hand pain, Presenteeism, WAI

## Abstract

**Background:**

Knowledge of factors associated with chronic pain is necessary for preventive strategies. The present study investigates biopsychosocial differences, with specific focus on rate of force development (RFD) and work ability, between workers with and without chronic upper limb pain.

**Methods:**

Eighty-two male slaughterhouse workers, 49 with chronic upper limb pain and 33 pain-free controls participated in the study. Maximal muscle strength, RFD, and muscle activity was determined from fast and forceful maximal voluntary contractions for the shoulder and hand. Participants filled out a questionnaire on work ability (work ability index), work disability (Work module of DASH questionnaire), fear avoidance, and self-rated health. Additionally, pressure pain threshold (PPT) was measured in muscles of the arm, shoulder and lower leg.

**Results:**

Muscle strength and RFD (determined within time intervals of 30, 50, 100, and 200 ms relative to onset of contraction) was 28 % and 58–78 % lower, respectively, in workers with chronic pain compared with pain-free controls, and paralleled by reduced muscle activity (all *p* < 0.001). Workers with chronic pain had lower PPT of the arm, shoulder and lower leg (*p* < 0.01), and reported impaired work ability index score and general health along with higher work disability and fear avoidance compared with controls (all *p* < 0.0001). No differences were observed between the groups in regard to age, BMI, physical activity level, job position and duration of slaughterhouse work (all *p* > 0.4).

**Conclusions:**

Chronic upper limb pain was paralleled by reduced neuromuscular function of the shoulder and hand along with impaired work ability, work disability and general health. Future studies on chronic pain management at the workplace should carefully consider the biopsychosocial nature of pain when designing and implementing preventive strategies.

## Background

Pain in the upper limb is frequent among employees with repetitive and forceful job tasks [1, 2]. Slaughtering and meat processing work involve high loading intensities and cyclic repetitive muscle actions of the upper limb and thus implies an elevated risk of work-related musculoskeletal disorders [3, 4]. In line with this, we recently found among 645 Danish slaughterhouse workers a prevalence of pain in the shoulder, elbow and hand/wrist of 60, 40 and 52 %, respectively [1]. Additionally, 38 % of the workers reported work disability due to upper limb pain emphasizing the functional consequences of arm, shoulder and hand pain on daily work performance.

Pain may originate from activation of peripheral nociceptors due to tissue damage. However, when the perception of pain for some reason persists beyond the expected time for tissue healing, chronicity has occurred [5]. The subjective experience of chronic pain is the result of the transduction, transmission and modulation of sensory information, signifying the involvement of central mechanisms in the perception of pain [6]. Hence, general hyperalgesia, evidenced by reduced pressure pain threshold (PPT) in a non-painful part of the body, is present in many variants of chronic pain including carpal tunnel syndrome [7], fibromyalgia [8], chronic low back pain [9], and trapezius myalgia [10]. However, evidence of a central component to work related chronic pain in the upper limb is lacking.

Chronic pain negatively impacts muscle functioning, evidenced by impaired maximal force production, motor control and endurance in painful conditions compared with healthy controls [11–13]. Additionally, previous research on office workers with trapezius myalgia showed, in a cross-sectional design, a markedly lowered rapid force capacity and neural drive during the initial phase of a maximal voluntary contraction [14]. Rate of force development (RFD) is influenced by both neural and muscular factors, encompassing central efferent neural drive, muscle fiber size and architecture along with maximal muscle strength [15–19]. Overall considered, there seem to exist a neurogenic inhibitory mechanism that limits rate of force development of chronically painful trapezius muscles. In line with this, chronic pain is a multifactorial experience composed of a multitude of complex biopsychosocial interactions, and likewise functional capacity assessments in these individuals are determined by biological, psychological and social factors [20]. Fear avoidance (i.e. the belief that movement exacerbates pain) along with musculoskeletal pain itself are examples of psychological factors that can influence patients physical performance [20–22] and could in theory inhibit efferent neural motor drive during fast and forceful movements. It is however unknown to what extent chronic upper limb pain inhibit activation and function of shoulder and hand muscles in workers with heavy and repetitive manual job-tasks, and whether pain related beliefs are impaired as a consequence of these pains.

Work related chronic pain is often accompanied by an escalating imbalance between work demands and individual resources, consequently affecting work ability [23]. In line with this, workers exposed to highly repetitive and forceful exertion, lack of sufficient recovery, and awkward postures [24, 25] have an elevated risk of both impaired work ability and musculoskeletal disorders [26–28]. Additionally, impaired work ability has been associated with loss of productivity, sickness absence, early retirement and all-cause mortality [27, 29–32]. To effectively prevent aggravation of pain and its associated harms among workers with heavy manual labor, knowledge on the biopsychosocial consequences of upper limb chronic pain are needed.

The aim of the present study was to investigate possible differences in biopsychosocial factors, with specific focus on rate of force development and work ability, between slaughterhouse workers with and without chronic upper limb pain. We hypothesized that workers with pain were expected to have impaired neuromuscular function and lower work ability score compared with pain-free controls.

## Methods

### Study design

A cross-sectional study regarding biopsychosocial consequences of chronic pain was conducted among 82 male slaughterhouse workers in Denmark, Europe. The study was a part of a randomized controlled workplace trial that was approved by The Danish National Ethics Committee on Biomedical Research (Ethical committee of Frederiksberg and Copenhagen; H-3-2010-062) and registered in ClinicalTrials.gov (NCT01716767). All experimental conditions conformed to The Declaration of Helsinki and participants were informed about the content and purpose of the study and gave their written informed consent to participate.

### Participants

Eighty-two male slaughterhouse workers were recruited from two slaughterhouses in Denmark, Europe: 49 with chronic upper limb pain and 33 pain-free controls. The workers with chronic pain were recruited in relation to a randomized controlled trial concerning the effects of strength training or usual-care ergonomics on chronic pain and work disability [2, 33].

The two-phased recruitment process contained a brief screening questionnaire (June 2012) followed by a clinical examination and a more in-depth questionnaire (August 2012). 645 Danish slaughterhouse workers received the screening questionnaire of which 595 replied and 410 were interested to participate in the research project.

Initial inclusion criteria for participants in the chronic pain group were: 1) working at a slaughterhouse for a minimum of 30 h/week, 2) pain intensity in the shoulder, elbow/forearm, or hand/wrist of 3 or more on a 0–10 VAS scale during the last 3 months, 3) stating at least “some” work disability on a five-point scale: “not at all”, “a little”, “some”, “much” to “very much” when asked the question “During the last 3 months, did you have any difficulty performing your work due to pain in the shoulder, arm or hand?”. The inspiration for this single-item question came from the work module of the DASH questionnaire [34]. Of the 410 interested respondents, 145 met the above inclusion criteria for the chronic pain group and were invited for a clinical examination. Initial inclusion criteria for participants in the pain-free control group were: 1) working at a slaughterhouse for a minimum of 30 h/week, 2) pain intensity in the shoulder, elbow/forearm, or hand/wrist of 1 or less on a 0–10 VAS scale during the last 3 months, 3) stating “not at all” work disability scoring on the five-point scale mentioned above. Of the 410 interested respondents, 50 met the above inclusion criteria for the pain-free control group and were invited for a clinical examination.

A total of 178 employees (135 with chronic pain and 43 pain-free controls) were included for the clinical examination. Furthermore, at the day of the clinical examination participants filled in another questionnaire. The following inclusion criteria applied for participants in the chronic pain group: 1) pain intensity in the shoulder, elbow/forearm, or hand/wrist regions of at least 3 on a 0–10 VAS scale during the last week, 2) pain should have lasted more than 3 months, 3) frequency of pain of at least 3 days per week during the last week. The following inclusion criteria applied for participants in the pain-free control group: 1) pain intensity in the shoulder, elbow/forearm, or hand/wrist of 1 or less during the last week, 2) frequency of pain of 0 days per week during the last week.

**Fig. 1:**
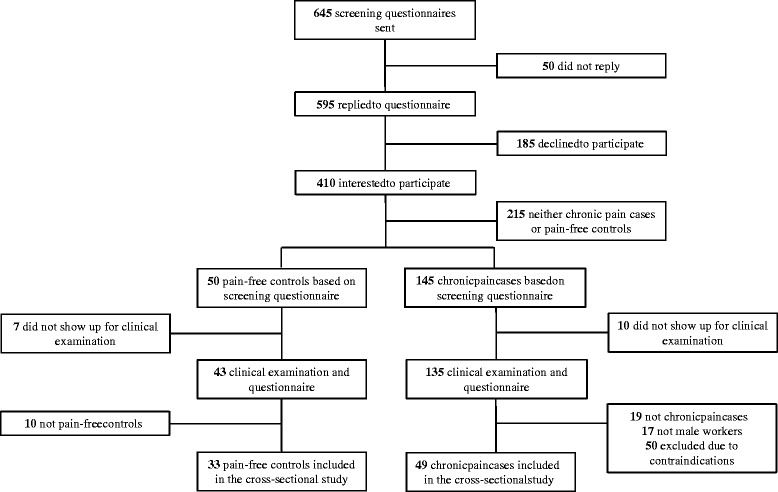
Flow of participants

Exclusion criteria for participants in both groups were: 1) recent traumatic injury of the neck, shoulder, arm or hand regions, 2) symptoms of carpal tunnel syndrome, 3) hypertension (Systolic BP > 160, diastolic BP > 100), 4) a medical history of cardiovascular diseases, 5) female worker. Participant flow and characteristics of participants are shown in Fig. 1 and Table 1, respectively.

**Table 1:** Demographics and pain intensity of study participants. Mean (SD)

	Chronic pain	Pain-free control
Demographics		
Age (years)	45 (11)	45 (11)
Height (cm)	179 (7)	178 (7)
Weight (kg)	90 (16)	87 (13)
Body Mass Index (kg/m^2^)	28 (5)	28 (5)
Weekly working hours	40 (1)	39 (6)
Duration of slaughterhouse work (years)	17 (10)	16 (13)
Number of participants	49	33
Clinical		
Shoulder pain intensity previous week (0-10)	5.6 (2.3)	0.1 (0.3)
Elbow/Forearm pain intensity previous week (0–10)	3.9 (2.7)	0.0 (0.2)
Hand/Wrist pain intensity previous week (0–10)	3.7 (2.9)	0.2 (0.6)
Pain duration > 3 months (%)	100	0

### Outcome measures

The following describes the experimental setup including the questionnaire and physiological test procedure. All outcome assessors were blinded to group allocation.

#### Muscle strength and rate of force development

Maximal voluntary contraction strength (MVC) was obtained during isometric muscle contractions performed for the shoulder- (external rotation) and wrist muscles (hand extension), using a custom-built dynamometer with 2 strain gauge load cells (KIS-2, 1 KN, Vishay Transducers Systems) [2]. During the hand extension MVC, subjects were seated upright in a chair with the elbow flexed at 90° while applying outward-directed force to a vertically orientated handlebar (dynamometer setting) positioned in front of them [2]. The anterior part of the forearm was supported by the dynamometer apparatus and allowed the participants to perform maximal isometric MVC’s. Maximal shoulder muscle strength (MVC) was assessed during concurrent isometric external rotation of the gleno-humeral joint. During the MVCs, participants were instructed to press as fast and hard as possible [35]. Verbal encouragement and online visual feedback of the force exerted were given during both trials. The trial with the highest peak force was used for the subsequent analysis. Rate of force development (RFD, expressed in N/s) was determined during the early time intervals of rising muscle force (0–30, 0–50, 0–100 and 0–200 ms from onset of contraction) [17].

#### EMG processing and data analysis

Electromyography (EMG) signals were recorded from the extensor carpi radialis brevis during the hand extension MVCs and from infraspinatus during the shoulder MVCs. Electrodes were placed on the dominant side. A bipolar surface EMG configuration (Blue Sensor, Ambu A/S, Ballerup, Denmark) with an inter-electrode distance of 2 cm were used [36, 37]. Before affixing the electrodes, the skin of the respective area was shaved and prepared with scrubbing gel (Acqua gel, Meditec, Parma, Italy) to effectively lower the impedance. Electrode placements followed SENIAM recommendations (http://www.seniam.org). The electrodes were connected through thin shielded cables to a data-logger (Nexus10, Mind Media, Netherlands) that was placed in a flexible belt to ensure mobility during the testing procedure. EMG signals were sampled at 1,024 Hz. To ensure quality of the EMG signals, all recorded signals were visually inspected. Subsequent data filtering and data analysis was performed with custom-made Matlab programs (MathWorks).

During later off‐line analysis, all raw EMG signals obtained during the MVC trial was digitally high-pass filtered using a Butterworth 4th order high‐pass filter (10 Hz cutoff frequency). For each individual muscle (i.e. extensor carpi radialis brevis and infraspinatus), a moving root-mean-square (RMS; 500-ms time constant) routine was used to smooth the EMG signals and to identify peak EMG amplitude [38]. Additionally, rate of EMG rise (RER) was calculated as the slope of the filtered EMG signal during the rising phase of the EMG-time curve for all time intervals (0–30, 0–50, 0–100 and 0–200 ms relative to onset of EMG rise).

#### Work ability

Work ability was rated by the work ability index questionnaire (WAI) [39]. Based on the answers of the seven items, an index score ranging from 7–49 was calculated and further classified into four categories: 7–27 (poor work ability), 28–36 (moderate work ability), 37–43 (good work ability) and 44–49 (excellent work ability) [33, 39].

#### Pain intensity

Pain intensity, experienced during the last 7 days, was rated subjectively using the 0–10 modified VAS scale, where 0 indicates “no pain at all” and 10 indicate “worst pain imaginable” [40, 41]. The shoulder, elbow/forearm and hand/wrist regions were defined by drawings from the Nordic questionnaire [42].

#### Work disability

Participants rated work disability by the work module of the Disability of the Arm Shoulder and Hand (DASH) questionnaire: *“Select which best describes your physical ability in the past week. Did you have any difficulty…1) Using your usual technique for your work? 2) Doing your usual work because of arm, shoulder or hand pain? 3) Doing your work as well as you would like? 4) Spending your usual amount of time doing your work?”* Participants replied on a 5-point Likert scale from ‘no difficulty’ to ‘unable’. For comparability with VAS pain scores, the work disability score was normalized on a scale of 0–100, where 100 represents the highest level of disability [34].

#### Self-rated health

Self-rated health was evaluated with the single global health-rating item from the Medical Outcomes Survey 36 item short form (SF-36) questionnaire [43, 44]. Participants responded to the question “How do you rate your overall current health?” on a 5-point Likert scale ranging from 1 (excellent) to 5 (poor).

#### Fear avoidance

Fear avoidance was evaluated using a tailor-made single-item question before and after the intervention period. Participants responded to the question: “Fast and forceful arm movement exacerbates pain in my shoulder, arm or hand?” on a 4 point Likert scale with the response options not at all (1), a little (2), some (3), a lot (4). This question was included because slaughterhouse work tasks commonly involve fast and forceful movement of the arm, shoulder and hands.

#### Pressure pain threshold

Pressure pain threshold (PPT) was assessed in the painful muscles of the shoulder and arm (infraspinatus and extensor carpi radialis brevis) and a nonpainful reference muscle (tibialis anterior) using an electronic pressure algometer (Somedic Productions AB, Sollentuna, Sweden, Europe). Pressure was manually applied perpendicular to the skin at the mid-belly of the 3 muscles at a rate of 30 kPa.s-^1^, and the contact area of the circular probe was 1 cm^2^ [10]. The participant was not aware of the reading of PPT on the display, and was instructed to push the patient operated switch on a pinch handle mounted on the algometer when the sensation of “pressure” changed to “pain.” PPT was measured 3 times at the infraspinatus, extensor carpi radialis brevis and tibialis anterior with 1½ min between each measurement alternating between the 3 muscles [10]. PPT for each muscle was subsequently expressed as the average value of the 3 measurements. Previous studies have shown satisfactory to good test-retest reliability of PPT [45, 46].

### Power calculation

The sample size calculation was based on the work ability index score (SD 4.8). If the true difference between the means in the chronic pain and pain-free control is 5, we will need to study 34 subjects with chronic pain and 17 pain-free control subjects to be able to reject the null hypothesis that the population means of the experimental and control groups are equal with probability (power) 0.8. The Type I error probability associated with this test of this null hypothesis is 0.01.

### Statistical analysis

Statistical analyses were performed using SAS version 9.2 (SAS Institute, Cary, NC). Because there were only two comparison groups at a single time-point, between-group comparisons (chronic pain vs pain-free controls) for the main variables were evaluated using unpaired t-testing. We used Bonferroni correction for multiple correlated end-points [47] to adjust the critical p-value, which then was set to 0.01. Results are given as least square means and standard error (SE) unless otherwise stated. An alpha level of 0.01 or less was accepted as statistically significant.

## Results

Table 1 shows demographics of the study population. No significant differences were observed for age, weight, BMI, level of physical activity, duration of slaughterhouse work, or job position (meat cutter, meat packer, slaughter) between the two groups (all *p* > 0.4), whereas pain intensity of the shoulder, arm and hand was significant higher in the chronic pain group compared with the pain-free controls (*p* < 0.0001). Two participants in the chronic pain group did not perform the MVC tests. In the control group, the hand extension and shoulder rotation MVC’s were not performed by six and eight participants, respectively. Missing EMG data from the MVC’s were present in 0–6 % of the participants.

**Fig. 2:**
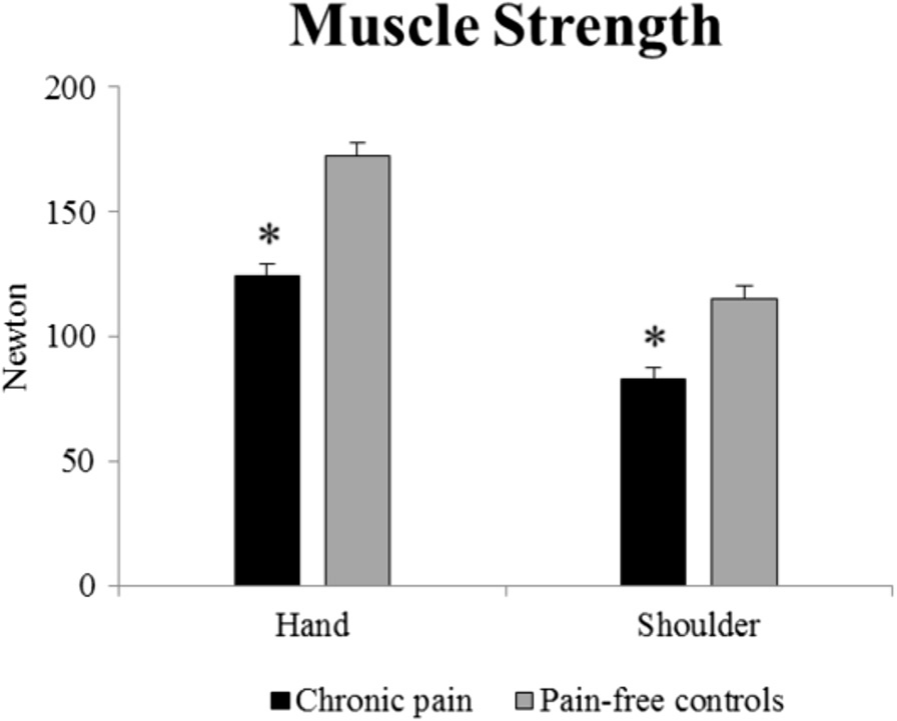
Maximal muscle strength. Maximal voluntary contraction strength in workers with chronic pain and pain-free controls. A custom made dynamometer was used to measure maximal voluntary contraction strength (MVC) during isometric contractions performed for the shoulder- (external rotation) and wrist muscles (hand extension). Mean (SE). * denotes statistical different from pain-free controls

**Fig. 3:**
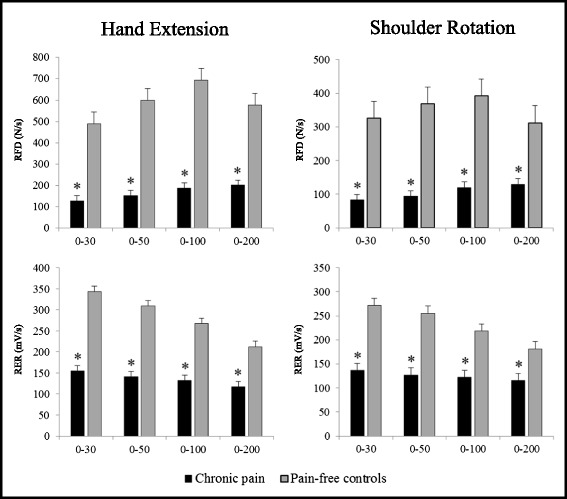
Rate of force development. Differences in contractile rate of force development (RFD) and rate of EMG rise (RER) for different time-intervals during hand and shoulder MVCs in workers with chronic pain and pain-free controls. A custom made dynamometer was used to measure maximal voluntary contraction strength (MVC) during isometric contractions performed for the shoulder- (external rotation) and wrist muscles (hand extension). Surface EMG was obtained from extensor carpi radialis brevis (during hand extension) and infraspinatus (during shoulder rotation). Mean (SE). * denotes statistical different from pain-free controls

There was a significant difference between groups for peak force and RFD during both shoulder rotation and hand extension (all *p* < 0.001). Shoulder rotation and hand extension strength was 28 % lower in participants with chronic pain compared to pain-free controls (Table 2; Fig. 2). RFD was 58–78 % lower for all time intervals and contraction modes in the chronic pain group compared to pain-free individuals (Table 2; Fig. 3).

**Table 2:** Differences in neuromuscular performance

	Chronic pain	Pain-free Control	Between group difference	P-value
Muscular function				
Hand strength (N)	124 (112 to 136)	173 (161 to 184)	−48 (−66 to −31)	<.0001
Shoulder strength (N)	83 (75 to 90)	115 (100 to 131)	−33 (−48 to −18)	0.0003
Hand extension RFD				
0–30 (N/s)	128 (85 to 171)	489 (389 to 589)	−361 (−454 to −269)	<.0001
0–50 (N/s)	154 (101 to 206)	599 (485 to 713)	−446 (−554 to −337)	<.0001
0–100 (N/s)	187 (128 to 146)	695 (581 to 808)	−507 (−621 to −394)	<.0001
0–200 (N/s)	201 (151 to 252)	576 (500 to 651)	−374 (−460 to −288)	<.0001
Shoulder rotation RFD				
0–30 (N/s)	82 (55 to 110)	325 (193 to 458)	−243 (−343 to −143)	0.001
0–50 (N/s)	94 (62 to 125)	368 (227 to 509)	−274 (−382 to −167)	0.0006
0–100 (N/s)	119 (82 to 157)	393 (27 to 51)	−273 (−372 to −174)	0.0001
0–200 (N/s)	129 (95 to 163)	312 (232 to 393)	−183 (−256 to −110)	0.0001
Hand extension RER				
0–30 (mV/s)	155 (113 to 197)	343 (295 to 392)	−188 (−252 to −124)	<.0001
0–50 (mV/s)	141 (107 to 176)	309 (273 to 345)	−168 (−218 to −117)	<.0001
0–100 (mV/s)	132 (106 to 158)	267 (247 to 288)	−135 (−171 to −100)	<.0001
0–200 (mV/s)	117 (98 to 136)	212 (198 to 226)	−95 (−121 to −70)	<.0001
Shoulder rotation RER				
0–30 (mV/s)	137 (101 to 172)	271 (223 to 320)	−135 (−193 to −77)	<.0001
0–50 (mV/s)	127 (94 to 160)	255 (215 to 296)	−128 (−180 to −77)	<.0001
0–100 (mV/s)	122 (95 to 149)	218 (195 to 243)	−96 (−134 to −59)	<.0001
0–200 (mV/s)	116 (95 to 136)	182 (167 to 197)	−66 (−93 to −39)	<.0001

Differences in muscle strength, contractile rate of force development (RFD) and rate of EMG rise (RER) for different time-intervals during hand and shoulder MVCs in workers with chronic pain and pain-free controls. A custom made dynamometer was used to measure maximal voluntary contraction strength (MVC) during isometric contractions performed for the shoulder- (external rotation) and wrist muscles (hand extension). Results from surface EMG measurements of extensor carpi radialis brevis (during hand extension) and infraspinatus (during shoulder rotation) are also shown in the table. Mean (95 % CI)

There was a significant group difference for peak EMG and RER during shoulder and hand MVC (all *p* < 0.0001). Infraspinatus peak EMG was 34 % lower, and extensor carpi radialis brevis peak EMG was 24 % lower in the chronic pain group during shoulder rotation and hand extension, respectively, compared with the control group. Likewise, RER was 36–55 % lower for all time intervals in the chronic pain group during all contraction modes (Table 2; Fig. 3).

**Fig. 4:**
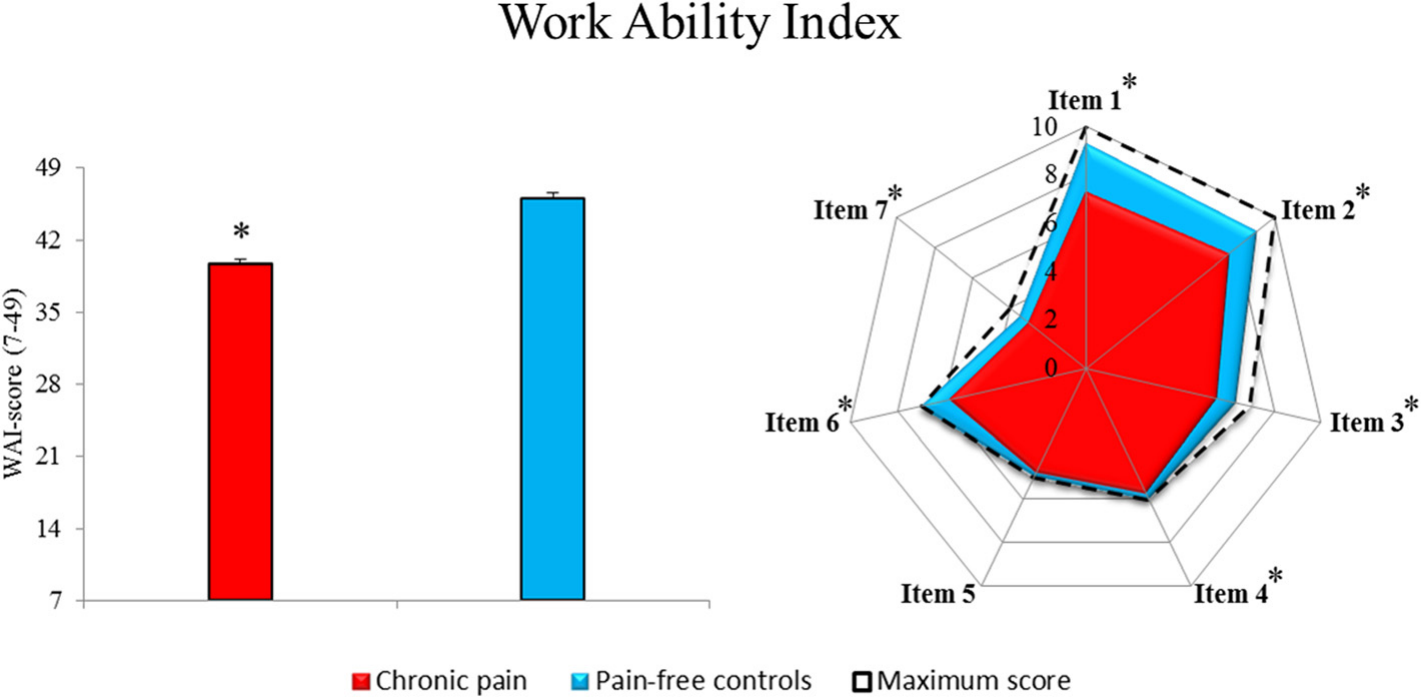
Work ability index. Illustration of between group differences in work ability index score (left side), and work ability single item score (right side). Item 1) Current work ability compared with the lifetime best, 2) Work ability in relation to the demands of the job, 3) Number of current diseases diagnosed by a physician, 4) Estimated work impairment due to diseases, 5) Sick leave during the past year, 6) Own prognosis of work ability two years from now, 7) Mental resources. Dotted line represents the maximum score possible for the single items of the work ability index. * denotes statistical different from pain-free controls

Significant group differences were observed for work ability, DASH work module, fear avoidance and self-rated health (all *p* < 0.0001). Work ability (WAI score) and self-rated health was lower, whereas work disability (DASH work) and fear avoidance was higher in participants with chronic pain compared to pain-free controls (Table 3, Fig. 4). All WAI single item scores, except for item 5 (sick leave during the past year; *p* = 0.27), were significantly lower in workers with chronic pain compared to pain-free controls (all *p* < 0.01; Table 3, Fig. 4). Additionally, PPT for both tibialis anterior, extensor carpi radialis brevis and infraspinatus was 21, 31 and 24 % lower, respectively, in workers with chronic pain compared with pain-free controls (Table 3).

**Table 3:** Work-related and clinical differences

	Chronic pain	Pain-free Control	Between group difference	p-value
Work-related				
DASH Work-module (0–100)	28 (23 to 33)	0 (0 to 0)	28 (22 to 34)	<.0001
Work Ability Index (7–49)	39.7 (38.4 to 40.9)	46.1 (45.3 to 46.9)	−6.4 (−8.1 to −4.8)	<.0001
Item 1: Current work ability compared with the lifetime best (0-10)	7.3 (6.9 to 7.7)	9.3 (9.0 to 9.6)	−2.0 (−2.6 to −1.5)	<.0001
Item 2: Work ability in relation to the demands of the job (2–10)	7.6 (7.2 to 8.0)	9.0 (8.6 to 9.5)	−1.4 (−2.0 to −0.9)	<.0001
Item 3: Number of current diseases diagnosed by a physician (1–7)	5.6 (5.1 to 6.0)	6.4 (6.0 to 6.7)	−0.8 (−1.4 to −0.2)	0.0047
Item 4: Estimated work impairment due to diseases (1–6)	5.7 (5.5 to 5.9)	6.0 (6.0 to 6.0)	−0.3 (−0.6 to −0.1)	0.0003
Item 5: Sick leave during the past year (1–5)	4.7 (4.5 to 4.9)	4.9 (4.7 to 5.1)	−0.2 (−0.5 to 0.2)	0.2682
Item 6: Own prognosis of work ability two years from now (1–7)	5.8 (5.3 to 6.3)	7.0 (7.0 to 7.0)	−1.2 (−1.8 to −0.6)	<.0001
Item 7: Mental resources (1–4)	3.0 (2.8 to 3.2)	3.5 (3.3 to 3.7)	−0.5 (−0.8 to −0.1)	0.0036
Clinical				
Self-rated health (1–5)	3.0 (2.8 to 3.2)	1.7 (1.4 to 2.0)	1.3 (1.0 to 1.6)	<.0001
Fear Avoidance (1–4)	2.6 (2.4 to 2.8)	1.1 (1.o to 1.1)	1.5 (1.2 to 1.8)	<.0001
PPT tibialis Anterior (kPa)	805 (701 to 910)	1014 (900 to 1130)	−210 (−342 to −78)	0.0020
PPT extensor Carpi Radialis Brevis (kPa)	639 (554 to 725)	918 (824 to 1013)	−279 (−387 to −171)	<.0001
PPT infraspinatus (kPa)	573 (494 to 652)	753 (666 to 839)	−180 (−280 to −81)	0.0004

Work ability index score, work disability (DASH-work module), self-rated health, fear avoidance and pressure pain threshold (PPT) in workers with chronic pain and pain-free controls. Mean (95 % CI)

## Discussion

The present study showed that workers with upper limb chronic pain demonstrate depressed neuromuscular function of the shoulder and hand along with impaired work ability, work disability and general health compared with pain-free controls. Obviously differences in pain intensity and pain duration existed between workers with chronic pain and pain-free controls. However, no differences were observed between the groups in regard to age, BMI, physical activity level, job position and duration of slaughterhouse work, suggesting that other factors seem to influence why some workers experience work related chronic pain. Therefore it is of clinical interest to investigate the mechanisms underlying chronic upper limb pain and disability in workers with manual labor.

Maximal muscle strength and neuromuscular activity was reduced by 28 % and 24–34 %, respectively, in the chronic pain group compared to the pain-free controls (Table 2). This illustrate that activation of chronically painful upper limb muscles is specifically inhibited during high force contractions. However, the ability to rapidly exert force (i.e. RFD) was reduced by 58–78 % and paralleled by reduced muscle activity during the rising phase of muscle force (i.e. RER). Previous research on office workers with trapezius myalgia showed, in a cross-sectional design, a markedly lowered rate of force development and reduced neural drive during the initial phase of a maximal voluntary contraction compared with healthy controls [14]. The present study confirms that maximal muscle strength is less affected than rate of force development in chronically painful muscles, however this study is the first to show this in shoulder and hand muscles of workers with physical demanding manual work. The marked reduction in rate of EMG rise observed in the chronic pain group, especially in the very initial contraction phase (0–50 ms), suggest that neural adaptation mechanisms may be strongly responsible for the deficit in RFD. Lowered rate of EMG rise might be explained by reduced motor neuron firing frequency and/or recruitment of high-threshold motor units in the very initial phase of contraction [15, 48]. However, the reduction in RFD was greater than the reduction in EMG (RER), proposing that the debilitating effect of muscle pain on rapid force capacity could also involve muscular factors such as muscular and viscoelastic atrophy. In addition, the maximal voluntary muscle contractions could in itself have aggravated pain among the workers with chronic pain, which could have lowered RFD and stopped the contraction before maximal force was reached.

General hyperalgesia, evidenced by reduced PPT in the non-painful muscle of the leg, was observed among the workers with chronic pain. This overall increase in sensitivity to pressure could reflect central sensitization, which is a phenomenon where nociceptive inputs can trigger excitatory synaptic responses and depressed inhibition of central nociceptive circuits, causing amplified reactions to noxious inputs [49–51]. The possible existence of central sensitization in workers with chronic upper limb pain further advocates the presence of neural alterations elicited by the persistence of pain over time, underlining the chronicity of these pains. As chronic pain is a multifactorial experience composed of complex biopsychosocial interactions, functional capacity assessments in these individuals are likewise determined by both biological, psychological and social factors [20]. Thus it could be speculated, that pain related beliefs, such as fear avoidance (i.e. the belief that fast and forceful movements exacerbated pain) might have affected the performance during the fast and forceful MVC’s. Thus, pain related inhibitory feedback mediated through high force excitation of golgi organs (by high group IV afferent activity leading to increased Ib inhibitory interneuron activity in the spinal cord) may have the potential to limit neural drive and decrease maximal force output during the high force phase of isometric contractions [52]. However, this reflex arc likely have limited influence on spinal motoneuron activation during the very early phase of rising muscle force, where instead, feed-forward mechanisms, such as fear avoidance, may have the potential to limit motor output and thus neural drive [14]. In the present study we found enhanced fear avoidance (i.e. that fast and forceful arm movement exacerbates pain) in the group of workers with chronic pain compared to pain-free controls. Thus, it seems possible that psychological pain related beliefs could have influenced initial muscle activation and thus rate of force development, in workers with chronic pain.

The results are likely to have a significant functional impact on the repetitive and forceful arm, shoulder and hand motions during slaughterhouse work possibly by weakening joint stability, motor control and precision during cutting and tearing tasks. This is additionally supported by the higher work disability score, as assessed by the work module of the DASH questionnaire, among the workers with chronic pain. Hence, chronic pain was paralleled by functional impairment of the arm, shoulder and hand during daily work tasks indicating an imbalance between individual capacity and work demands. This is further acknowledged, by the observed reduction in work ability (i.e. lowered WAI-score) in the chronic pain group (Fig. 4). The concept of work ability reflects the relation between capacity of the worker and demands of the work, and takes into consideration both demands of the work, health status, and physical and mental resources [53, 54]. As a multidimensional instrument, work ability (index) has been related to musculoskeletal pain, chronic disease, productivity, sickness absence, early retirement and all-cause mortality [29–32, 55]. Likewise, workers exposed to highly repetitive and forceful exertion, lack of sufficient recovery, and awkward postures [24, 25] have an elevated risk of both impaired work ability and musculoskeletal disorders [26–28]. Despite lower work ability in the group with chronic pain observed in the present study, ther index score of 39.7 was still categorized as good. Additionally, when analyzing the items of the WAI separately, no difference in item 5, regarding sick leave during the past year existed between the groups (Fig. 4). Thus, in this group of workers, chronic upper limb pain is paralleled with self-reported decreased work productivity, evidenced by impaired work performance and work ability (DASH-W and WAI) without having direct consequences on sick leave. Consequently, in the present study, chronic pain seems to foster presenteeism (i.e. decreased on the-the-job performance due to health problems) while not leading to absenteeism, which is further acknowledged by the fact that the group with chronic pain were active on the labor market working fulltime at the slaughterhouse [56]. It should however be mentioned, that the differences in DASH and WAI score could potentially be attributed to the inclusion criteria, as participants in the two groups were selected to be different in perceived work disability.

The biopsychosocial model consider chronic pain and disability as the consequences of the dynamic interplay between physiological, psychological, and social factors [6]. In the present study, the chronicity of pain among this group of workers was established by self-reported inclusion criteria and further objectively recognized by an observed parallelism between pain and indicators of neural alterations (central sensitization). Besides obvious biological deficiencies, such as reduced strength and rapid force capacity, also impaired psychosocial features were present in the participants with chronic pain (fear avoidance and WAI item 7 on mental resources). The interaction of the abovementioned factors could have led to an imbalance between individual capacity and work demands, as evidenced by impaired work ability and work disability, consequently affecting overall health (i.e. self-rated health) among the workers with chronic pain. Self-rated health is a major independent predictor of objective health, morbidity and mortality [57–59] and especially symptoms such as chronic pain and fatigue are important constituents of self-rated health. Additionally, chronic pain is independently and significantly related to self-rated health [60] and impaired general health is associated with poor recovery from chronic pain [61]. Future studies on upper limb chronic pain management must carefully consider the biopsychosocial nature of pain in order to augment treatment success.

### Strength and limitations

Our study has both strengths and limitations. Combining direct measures of pressure pain threshold and rapid force capacity with subjective reporting of pain and its biopsychosocial consequences is a strength. A study limitation was that no muscle biopsies were obtained to examine intramuscular differences potentially accountable for the observed reduction of rapid force capacity and maximal muscle strength. Finally, the exclusion and inclusion criteria used in this cross-sectional study confines the generalizability of the results to individuals with chronic upper limb pain exposed to highly repetitive and forceful job tasks.

## Conclusions

Chronically painful upper limb muscles demonstrated impaired rapid force capacity and neural activation compared to non-painful muscles. Further, workers with chronic pain showed impaired work ability, work disability and general health compared to pain-free controls. Future studies on rehabilitation of upper limb chronic pain in workers with physically demanding job tasks must carefully consider the biopsychosocial nature of pain in order to augment treatment success.

## Abbreviations

DASH: Disability of the Arm, Shoulder and Hand questionnaire
EMG: Electromyography
MVC: Maximal Voluntary Contraction
PPT: Pressure Pain Threshold
RER: Rate of EMG Rise
RFD: Rate of Force Development
VAS: Visual Analog Scale
WAI: Work Ability Index
